# Type D Personality as a Risk Factor in Coronary Heart Disease: a Review of Current Evidence

**DOI:** 10.1007/s11886-018-1048-x

**Published:** 2018-09-12

**Authors:** Nina Kupper, Johan Denollet

**Affiliations:** 1 Center of Research on Psychology in Somatic Diseases, Tilburg University, PO box 90153, 5000 LE Tilburg, The Netherlands; 2 Department of Medical & Clinical Psychology, Tilburg University, Tilburg, The Netherlands

**Keywords:** Type D personality, Coronary heart disease, Heterogeneity, Risk profiles, Atherosclerosis, Cardiac mortality, Stress, Biobehavioral mechanisms

## Abstract

**Purpose of the Review:**

This review article synthesizes recent research findings on the psychological context of Type D personality and the mechanisms through which Type D affects disease progression and prognosis among patients with coronary heart disease (CHD).

**Recent Findings:**

One in four patients with CHD has a Distressed (Type D) personality, which is characterized by two stable traits: social inhibition and negative affectivity. Type D personality predicts increased mortality and morbidity burden, and poorer health-related quality of life. Type D is part of a family of psychosocial risk factors that affect CHD prognosis. The pattern of co-occurrence of these psychosocial factors and intra-individual differences in psychosocial profiles may affect risk prediction accuracy. Multiple biological and behavioral processes have been associated with Type D personality.

**Summary:**

Identifying pathways explaining the observed associations between Type D personality and CHD is important to improve etiological and pathophysiological knowledge and to design personalized interventions, and targeting specific risk-associated pathways.

## Introduction

Type D, or *Distressed*, personality is an established risk factor for the development and prognosis of coronary heart disease (CHD) [1, 2], and since 2012, Type D has been included in the European Cardiovascular Prevention guideline as a risk factor to screen for [2]. Type D personality is characterized by a combination of social inhibition and negative affectivity and occurs in approximately one in four patients with CHD. Due to improved treatment options [3], CHD has become a long-term chronic condition, putting a high burden on patients’ quality of life [4], caregivers, and the health care system at large [5]. Psychosocial risk factors, among which is Type D personality, are prevalent in patients with heart disease and have shown to diminish patients’ well-being even more [6]. Risk prediction studies estimating the increased risk associated with Type D personality have shown considerable heterogeneity [1], just is the case for other (psychosocial) risk factors [7–10]. Moreover, (psychosocial) risk factors most often do not occur in isolation, but rather cluster together within patients, complicating risk assessment. Prior research has acknowledged the association of Type D personality with depressive symptoms, anxiety, and chronic stress [11–13]. However, research has been focusing predominantly on independent effects, ignoring the natural order and clustering of psychological constructs [14•], i.e., from stable predispositions (traits) to more episodic factors (e.g., depression, chronic stress) and states (e.g., mood, acute stress) [15]. This review will argue that Type D personality should be viewed in this psychological context, and that research is needed to identify the psychological mechanistic processes in addition to investigating independent effects of selected sub-elements of that process.

Biological processes and health-related behaviors play a role in the association between Type D personality and CHD progression. Adherence to medication, lifestyle modification, and post-event cardiac rehabilitation are at the forefront of treatment to prevent disease progression during the long-term follow-up of patients with CHD [2]. These behavioral pathways may be affected by a patient’s predisposition, e.g., Type D personality, thereby affecting cardiac prognosis. In addition to these indirect behavioral pathways, direct biological pathways may be identified, with stress-related processes affecting pathophysiological processes, thereby influencing disease progression and prognosis. It is through understanding these mechanisms that new and personalized interventions may be developed, taking within-person risk factor profiles into account.

In the current review, we thus will summarize sources of heterogeneity in outcome prediction as well as recent research findings on the psychological context of Type D personality. Then, we will synthesize recent findings on biological and behavioral mechanisms through which Type D affects disease progression and prognosis among patients with CHD.

## Type D Personality and the Psychosocial Risk Spectrum

Distressed (or Type D) personality combines two stable traits, i.e., *negative affectivity*, or the tendency to experience negative emotions (e.g., anger, sadness, fear, irritability) across time and situations, together with the trait *social inhibition*, which refers to the tendency not to share these emotions in social interactions, because of fear of rejection or disapproval [16]. Type D personality is stable across time and has a substantial underlying genetic component [17], and established cross-cultural measurement equivalence [18]. Type D personality is a vulnerability factor for future episodes of emotional stress such as depressive episodes [19, 20] and anxiety [20].

Heterogeneity in clinical outcome may derive from relatively stable individual differences in personality [21]. Personality characteristics affect the propensity to experience post-event emotional stress and may be instrumental in the preference for certain appraisal and coping styles. Indeed, Type D personality has previously been associated with heightened levels of emotional stress (e.g., anxiety, depression) [11, 12, 22, 23], as well as a preference for maladaptive coping styles [24, 25]. A recent study of our group in patients with CHD revealed a within-person clustering of social inhibition and negative affectivity with low resilience, high neuroticism, and introversion, and moderate levels of a variety of coping strategies [14•]. This suggests that this *high distress* group tried to deal with their stress in several ways, nonetheless experiencing high levels of anxiety and depression and reduced positive mood 6 months later [14•]. The individuals in this *high distressed* cluster were more often single and employed. A similar study showed that individuals in the high distress cluster (based on the European Society of Cardiology psychosocial screening instrument [2, 26]; which contained depression, tension and Type D) were younger, had a poorer lifestyle, and experienced more early adverse life events [27]. The intra-individual cluster approach exemplified in the two studies above is doing much more right to the complex nature of psychological functioning and its context, and as such using intra-individual clusters may improve risk prediction accuracy and provide new avenues for personalized interventions.

## Explaining Heterogeneity in Risk Prediction

The most recent meta-analysis on the prognostic effect of Type D personality [1] summarized findings of 12 individual studies (*N* = 5341). The study concluded that Type D personality imposed a significantly increased mortality risk (adjusted hazard ratio (Adj. HR) = 3.88 (95% CI, 2.58–5.85)) in patients with CHD, while there was no increased mortality risk in patients with heart failure (Adj. HR = 0.91 (95% CI, 0.66–1.27)). Importantly, cause of death often remains undetermined in patients with heart failure, forcing researchers to collapse all causes into one, all-cause category. Although the estimated heterogeneity in this meta-analysis was reduced by specifying the results per diagnostic subgroup, still substantial heterogeneity exists. This motivated the pursuit of a new analysis on existing data in CHD patients [28••], examining sources of heterogeneity in outcome prediction. Besides, the known effect of the stage of heart disease (i.e., coronary artery disease vs. heart failure) and the type of outcome measure (all-cause mortality vs. cardiac events and cardiac death), as well as the age of the participating patients, substantially explained variation in outcome prediction. Type D personality was particularly related to cardiac death and cardiac events, instead of non-cardiac death [28••], indicating the association of CHD-specific pathways with Type D personality, through which prognosis is affected. Also, it is important to realize in general that although studying all-cause mortality has practical advantages (e.g., easy to assess, no interpretation biases), it can dilute the significance of risk factors whose mechanistic pathways affect different, disease-specific causes of death. Recent studies reporting the un-relatedness of Type D with all-cause mortality [29, 30], as opposed to its relatedness with cardiac events and cardiac death [31], are in line with this reasoning.

## Behavioral Risk Pathways

Multiple studies testify to the role of various health behavioral aspects in explaining the increased incidence of heart disease and earlier progression of heart disease. In healthy, young [24], and older [32] samples as well as in patients with diabetes [33], research findings consistently relate Type D personality to less healthy eating behaviors. Also, across healthy and patient samples, Type D personality was associated with less regular physical exercise [23, 32–34]. Also in cardiac patients, the relationship between lack of exercise and Type D personality is evident, with Type D patients exercising less and feeling less motivated to exercise [23]. A third important health behavior that is impaired is treatment adherence. Patients with heart disease characterized by Type D personality demonstrate poorer medication adherence [35, 36], and self-management behaviors [14•, 36].

## Type D, Stress Hormones, Autonomic Activation, and the Etiology of Cardiovascular Disease

**Fig. 1:**
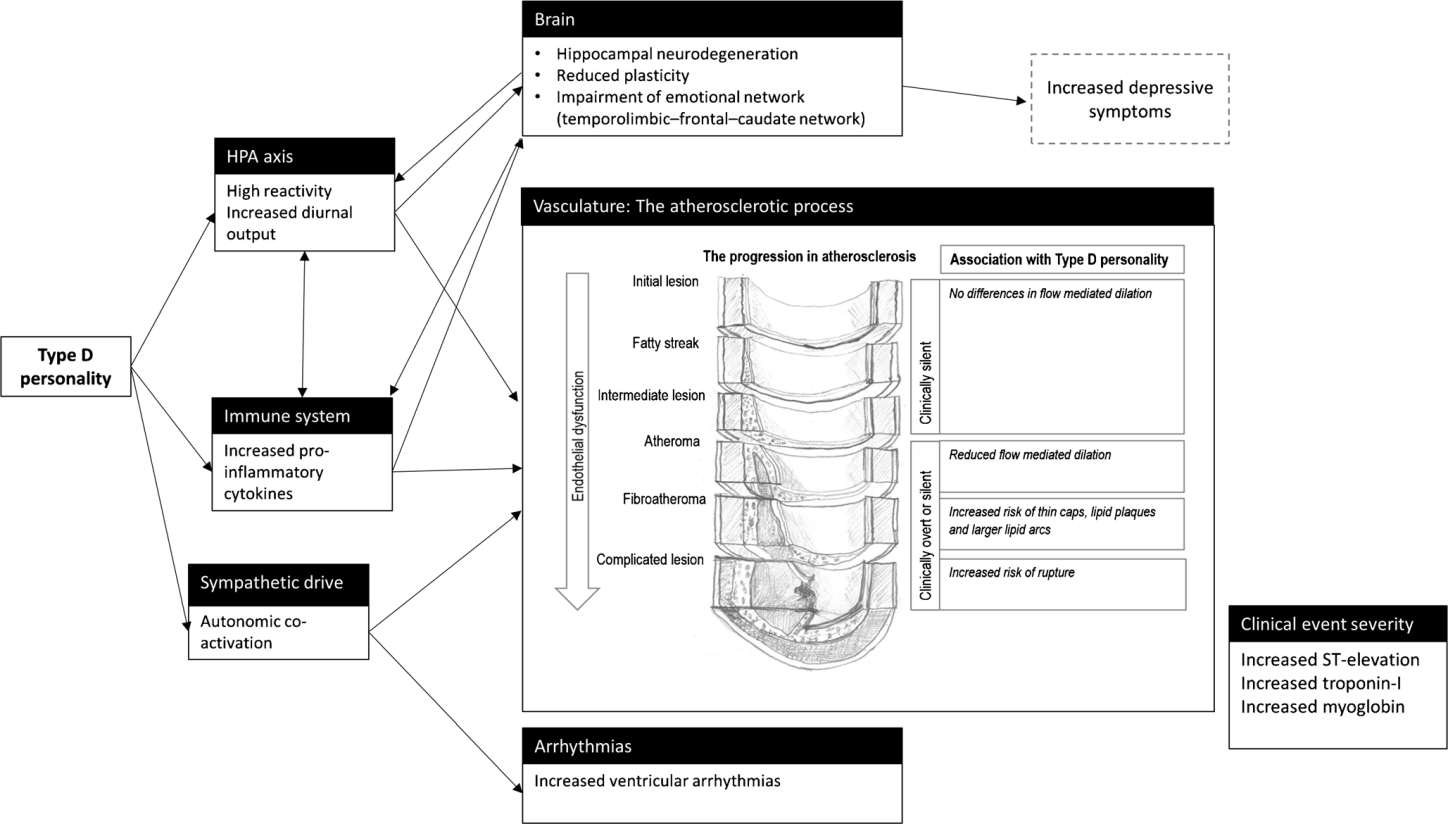
Pathophysiological pathways of Type D personality. Elements from this figure (i.e., the atherosclerotic process) were adapted from a figure at https://commons.wikimedia.org/wiki/File:Endo_dysfunction_Athero.PNG under the Creative Commons Attribution-Share Alike 3.0 Unported license [70]

The association of Type D personality with CHD-related outcomes can be understood by altered biological processes that accompany prolonged psychological distress. Having a *Distressed* personality may be considered a lifelong stressor, and thus may alter the functioning of the body’s stress hormones, i.e., cortisol and norepinephrine. As shown in Fig. 1, the two primary output pathways of the brain to the heart involve the neurohormonal system (the hypothalamus-pituitary-adrenal (HPA) axis and the sympatho-adrenal medullary (SAM) axis) and the autonomic nervous system. Cortisol is secreted from the HPA axis and controls the stress response and a number of important bodily functions, such as the modulation of the production of pro-inflammatory cytokines by cytotoxic t cells. As such, cortisol is an important potential mechanism in the promotion of chronic low-grade inflammation and of atherosclerosis. Healthy young adults with Type D personality seem to produce a larger cortisol response to acute social stress [37, 38]. With respect to the diurnal rhythm, findings suggest a progressive disruption of HPA axis functioning with age, with one recent study in the general population reporting somewhat higher (though non-significantly) 24-h levels of cortisol in Type Ds compared to those in controls [39]. In several samples of patients with acute coronary syndrome, however, diurnal cortisol output was significantly increased in those with Type D personality [40, 41]. There is growing evidence that cortisol influences a series of processes that are important in the etiology and pathophysiology of heart disease, such as obesity, LDL cholesterol levels, endothelial dysfunction, rises in blood pressure, and an increased pro-inflammatory state [42]. Also, cortisol has been related to subclinical atherosclerosis [43] and coronary calcification [44], possibly through affecting the availability of nitric oxide synthase (NOS), through inflammation [45]. Finally, high cortisol levels were predictive of cardiovascular death, in patients with and without a cardiac history [46, 47]. Taken together, the specific link of cortisol with cardiovascular etiology, pathophysiology, and cardiovascular mortality strengthens the notion that the progressively high cortisol levels in individuals with Type D personality might be an important catalyzer of cardiovascular risk. Chronically high cortisol levels also seem to stimulate neurodegeneration, promoting depressed mood [48]. Future research that combines cortisol assessment with measurements of endothelial dysfunction and atherosclerosis will help elucidate the pathways by which Type D personality adversely affects CHD progression.

Norepinephrine serves as a second important stress (neuro-) hormone, secreted from the SAM axis in response to stress, as well as serving as a neurotransmitter of the sympathetic part of the autonomic nervous system. However, in the general population, there is one study showing no Type D-associated differences in urinary 24-h norepinephrine [39], whereas no information about the association between Type D personality and norepinephrine are available in patients with CHD.

Studies measuring cardiac sympathetic drive do suggest Type D-associated alterations though. In healthy young adults undergoing an endurance stressor (i.e., cold pressor), it was shown that Type D individuals responded with an exaggerated blood pressure response, and male Type D individuals showed an autonomic co-activation pattern in which both parasympathetic and sympathetic nervous systems became more active. Also, sympathetic activation was exaggerated in comparison to women and non-Type Ds [49]. Such co-activation response profile has been previously associated with increased cardiovascular risk by promoting arrhythmias [50]. Differences in sympathetic cardiac drive were also apparent in a clinical study, in which Type D personality was independently associated with the occurrence of ventricular arrhythmias, which are considered markers of sympathetic dysregulation [51]. As the sympathetic nervous system is directly involved in controlling inflammatory processes both systemically and locally, directly at atherosclerotic lesions [52], the observed sympathetic hyperactivity could play a role in promoting atherosclerosis. Future studies are encouraged to examine this pathway.

Taken together, the above-described stress pathways may affect different but converging pathophysiological processes affecting cardiovascular risk. Future research is needed to focus on the mechanistic diversity to improve risk stratification.

## Type D and Pathophysiological Pathways Related to Cardiac Risk

The pathophysiology of CHD is characterized by progressive accumulation of lipids and fibrous elements in the large arteries, i.e., atherosclerosis, culminating in myocardial ischemia and infarction. Endothelial dysfunction is an early marker, indicating increased cardiovascular risk before angiographic or ultrasonic evidence of atherosclerotic plaque is present [53]. Atherosclerosis is an inflammatory process. Meta-analytic data suggest patients with Type D personality have heightened markers of pro-inflammatory activity [54]. Due to arterial inflammatory activation, circulating monocytes migrate into the arterial intima and transform into macrophages. There, in the atheroma, the macrophages phagocyte oxidized lipoproteins thus contributing to endothelial dysfunction [55]. The atherosclerotic process is self-perpetuating, as the macrophage-transformed foam cells produce pro-inflammatory cytokines that amplify the local inflammatory response, as well as reactive oxygen species, such as superoxide anions [56]. The foam cells also play a role in the thrombotic complications of atherosclerosis, as they play a role in degrading extracellular matrix by producing matrix metalloproteinases (MMPs). As a consequence, an atherosclerotic plaque may rupture [55]. Some plaques are more vulnerable than others. Lipid plaques in the arterial wall are prone to grow, and eventually rupture, when these sites have a thin cap fibroatheroma [57].

Several lines of research show that Type D personality can be linked to earlier and later phases in the atherosclerotic process (Fig. 1). This suggests that not only Type D personality plays a role in CHD progression, but also may affect the etiology of CHD. Evidence for this position comes among others from studies showing increased prevalence of hypertension in primary care patients with Type D personality [58], the increased presence of coronary artery plaques in individuals with Type D personality without a history of CHD [59], and the increased risk of having/developing CHD associated with Type D personality in the general population [60, 61].

With respect to the proposed atherosclerotic processes, one study in healthy community dwelling individuals showed that Type D personality was unrelated to flow-mediated dilation (a non-invasive measurement of endothelial dysfunction, and indirect assessment of nitric oxide (NO) functioning), or diameter, of the brachial artery [60]. In patients with more advanced phases of endothelial dysfunction, i.e., with overt CHD, Type D personality was associated with reduced flow-mediated dilation [62•], and thus impaired endothelial-dependent vasodilation.

CHD patients with Type D personality had higher macrophage superoxide anion production compared to their non-Type D counterparts [56], perpetuating the atherosclerotic process. Several other studies have examined a culprit arterial lesion in CHD patients by optical coherence tomography, showing Type D-associated differences in plaque vulnerability. One study in 152 patients with CHD showed that patients with Type D personality had significantly more vulnerable plaques, with a 4.5 times higher risk of a lipid plaque, a 3 times higher risk of a thin cap fibroatheroma, and a 2.5 times increased chance of plaque rupture [63•]. Another study examining culprit lesions showed that CHD patients with Type D personality more often had a lipid plaque in comparison to their non-Type D counterparts. Also, the lipid arc in Type D patients was significantly wider [64]. With respect to plaque outcome, again Type D personality was associated with increased presence of thin cap fibroatheroma and increased ruptures, independent of clinical covariates, including cholesterol, smoking, and CRP [64]. This latter study further showed that health behavior habits such as poor eating explained part of the association of Type D with plaque vulnerability [64].

Recently, CHD patients treated with a drug-eluting stent were examined for in-stent restenosis at 1 and 2 years after percutaneous coronary intervention for the index event. Results showed that patients with Type D personality had a more than doubled increased risk of in-stent restenosis, independent of clinical covariates [65]. Post hoc analyses showed that negative affectivity, and to a somewhat lesser extent social inhibition, was driving the association. As for an explanation, the development of neoatherosclerotic lesions in the vicinity of vulnerable plaque seems an important mechanism of in-stent restenosis [66], and combined with the observation that vulnerable plaque is present in patients with Type D personality [64, 67], a more frequent occurrence of neoatherosclerosis in patients with Type D personality seems plausible.

Cardiac events may vary in severity, due to various factors that still need to be explored in association with psychosocial risk factors. Recent results did show that patients with recurrent acute coronary syndrome and Type D personality had a more severe cardiac event, reflected in a higher prevalence of ST elevation and more damage to the myocardium, i.e., higher levels of troponin-I and myoglobin [68]. Future studies are needed to examine underlying pathophysiological processes associated with Type D.

## Conclusions and Future Directions

Type D personality is an established independent risk marker in patients with CHD, prospectively associated with worse clinical and patient-reported outcomes, although substantial heterogeneity exists between studies. Research findings have attributed large parts of this heterogeneity to the disease stage, specificity of the outcome measure, and sample age. It is important to consider the mechanistic pathways through which a risk factor is likely to exert its effect when choosing endpoints. Otherwise, it might erroneously dilute the importance of the risk factor. Moreover, Type D personality should also be evaluated in its overall psychosocial context. The use of intra-individual profiles in risk assessment, by means of a smart, multidimensional psychosocial screener therefore is indispensable.

Type D personality has been associated with a number of plausible biological and behavioral mechanisms through which heart disease incidence and progression is affected. As CHD is multifactorial by nature, it is likely that Type D exerts its effect through a cluster of interacting mechanisms, each contributing a small part. Poor health behaviors cluster in individuals with Type D personality [36], putting them at risk for future heart disease and earlier disease progression. The biological pathways constitute a network, rather than a chain of events, and should be approached as such. Future research should take this more comprehensive approach, evaluating the network of potential biological and behavioral pathways linking Type D personality to cardiac events and cardiac death, and the diversity in pathophysiological “routes” that can be taken.

At the moment, there is no psychological intervention for Type D personality. Nevertheless, stepwise psychotherapy to improve depressive symptoms was shown to be particularly beneficial for CHD patients with Type D personality [69••]. Interventions for psychological problems that affect the course of somatic illness should ideally focus on improving psychological functioning as well as slowing disease progression, for which knowledge of the psychobiological mechanisms is a prerequisite. Also, small steps should be taken. Experimental studies should first determine proof of principle and proof of mechanism, before continuing to randomized controlled trials. In the meantime, clinicians should explicitly promote cardiac rehabilitation for CHD patients with Type D personality, as it improves their mood and quality of life, as well as physical functioning.
